# A U-Shaped Dual-Frequency-Reconfigurable Monopole Antenna Based on Liquid Metal

**DOI:** 10.3390/ma15041599

**Published:** 2022-02-21

**Authors:** Peng Qin, Qian-Yu Wang, Jun-Heng Fu, Chun-Wei Li, Cheng-Lin Zhang, Tian-Ying Liu, Lin Gui, Jing Liu, Zhong-Shan Deng

**Affiliations:** 1 CAS Key Laboratory of Cryogenics, Technical Institute of Physics and Chemistry, Chinese Academy of Sciences, Beijing 100190, China; qinpeng17@mails.ucas.edu.cn (P.Q.); wangqianyu19@mails.ucas.edu.cn (Q.-Y.W.); fujunheng17@mails.ucas.edu.cn (J.-H.F.); lichunwei20@mails.ucas.edu.cn (C.-W.L.); zhangchenglin17@mails.ucas.edu.cn (C.-L.Z.); liutianying17@mails.ucas.edu.cn (T.-Y.L.); lingui@mail.ipc.ac.cn (L.G.); jliu@mail.ipc.ac.cn (J.L.); 2 School of Future Technology, University of Chinese Academy of Sciences, Beijing 100049, China; 3 Department of Biomedical Engineering, School of Medicine, Tsinghua University, Beijing 100084, China

**Keywords:** dual-frequency antenna, liquid metal, monopole, reconfigurable antenna, ultra-wideband antenna

## Abstract

This study presents a U-shaped dual-frequency-reconfigurable liquid-metal monopole antenna. Eutectic Gallium–Indium (EGaIn) was used as a conductive fluid and filled in the two branches of the U-shaped glass tube. A precision syringe pump was connected to one of the branches of the U-shaped tube by a silicone tube to drive EGaIn, forming a height difference between the two liquid levels. When the height of liquid metal in the two branches met the initial condition of *L*_1_ = *L*_2_ = 10 mm, and *L*_1_ increased from 10 mm to 18 mm, the two branches obtained two working bandwidths of 2.27–4.98 GHz and 2.71–8.58 GHz, respectively. The maximum peak gain was 4.00 dBi. The initial amount of EGaIn also affected the available operating bandwidth. When the liquid metal was perfused according to the initial condition: *L*_1_ = *L*_2_ = 12 mm, and *L*_1_ was adjusted within the range of 12–20 mm, the two branches had the corresponding working bandwidths of 2.18–4.32 GHz and 2.57–9.09 GHz, and the measured maximum peak gain was 3.72 dBi. The simulation and measurement data corresponded well. A series of dual-frequency-reconfigurable antennas can be obtained by changing the initial amount of EGaIn. This series of antennas may have broad application prospects in fields such as base stations and navigation.

## 1. Introduction

To meet the needs of wireless communication with diverse functions, it has become a common trend to apply multiple antennas in electronic device systems. However, the further development of multiple antennas is restricted by electromagnetic interference. The reconfigurable antenna, as a multifunctional antenna, is a potential candidate to solve the electromagnetic interference problem of multiple antennas in principle. This is owing to the reconfigurable antenna having various working modes including reconfigurable frequency, reconfigurable pattern, reconfigurable polarization, and a reconfigurable combination of the above-mentioned modalities.

The common reconfigurable antenna structure integrates active devices such as radio-frequency microelectromechanical systems (RF-MEMS) [1], *p*-type intrinsic *n*-type (PIN) diodes [2], and varactor diodes [3] to control the on–off of the circuit, realizing the switch between different functions. To pursue the superior working performance of the antenna, such as having a larger working bandwidth, wider beam bandwidth and more polarization modes, the number of RF switches and circuit complexity are increasing, which will result in the nonlinear problem and reduced efficiency.

Considering the issues of environmental protection and health, the frequently used liquid metals are gallium or gallium-based alloys, and their melting points are usually at or around room temperature. Gallium-based liquid metals (GBLMs) have the characteristics of high electrical conductivity, high thermal conductivity, good fluidity, deformability and repairability. As a new type of multifunctional electronic material, GBLMs have been used in the fields of thermal management [4], biomedicine [5], additive manufacturing [6], printed electronics [7], skin electronics [8] and other fields. In recent years, the conductivity and fluidity of GBLMs have been paid more attention to by more researchers [9], and they have been used in the field of high-frequency electromagnetic fields, especially for the design of new liquid reconfigurable antennas [10,11].

Different from the above-mentioned antennas with switching components via disassembling or assembling like building blocks to achieve structural reconstruction, the liquid-metal reconfigurable antennas can directly realize the reconstruction of the antenna through continuous movement or deformation. The nonlinear problem is avoided since there are no active switching components in liquid-metal reconfigurable antennas. Additionally, the liquid-metal reconfigurable antennas can more effectively eliminate electromagnetic interference in multiantenna systems [12,13].

Single-frequency-reconfigurable antennas based on liquid metal have been studied by many researchers. In 2010, Masahiro Kubo et al. [14] proposed a retractable microfluidic radiofrequency antenna in which liquid metal was used as the conductor, and Polydimethylsiloxane (PDMS) and Ecoflex ™ as the substrate were utilized in combination. The tensile strain of the antenna could be stretched up to 120%, and the resonant frequency tuned from 0.738 GHz to 1.53 GHz. A slot antenna with tunable frequency driven by the continuous electrowetting method was proposed in 2014 [15], which manipulated the displacement of the liquid-metal slug to realize the reconstruction of the slot antenna. The antenna changed its resonant frequency over a 15.2% bandwidth [15]. The strategy of electrochemically controlled capillarity to reconfigure the liquid-metal antenna was proposed in 2015 [16]. A bias direct-current voltage was used to reversibly control the Eutectic Gallium–Indium (EGaIn) in the capillary rather than a mechanical pump. Working frequency of the antenna could range from 0.66 GHz to 3.4 GHz, and the radiation efficiency in this range varied from 41% to 70% [16]. A microfluidically reconfigured wideband frequency-tunable monopole antenna was brought up in 2016 [17]. The reconfiguration of the antenna was achieved by actuating liquid metal with a micropump, combining this with a capacitive-coupling feeding mechanism. This antenna obtained a working bandwidth of 1.29 GHz to 5.17 GHz [17]. In 2017, Gregory H. Huff et al. [18] provided an embedded vascular antenna. The shape of the dipole was a sine curve with rotationally symmetric power series growth. The liquid metal moved after pressurization to change the length of the curve and realized the change of the antenna resonant frequency. In 2018, Syed Imran Hussain Shah et al. [19] studied a microfluidic frequency-reconfigurable Quasi-Yagi dipole antenna. The volume of liquid metal in the driving dipole element and the three directors was controlled by programmable pneumatic micropumps. The antenna frequency was continuously tuned at 1.8–2.4 GHz, and the gain was between 8.0 and 8.5 dBi. In the previous work of our laboratory, the principle of thermal expansion of liquid metal was used to design a frequency-reconfigurable monopole antenna [20]. This well-packaged antenna eliminated the dependence on electrolyte solutions and obtained a working bandwidth of 1.25–2.00 GHz. Additionally, a reconfigurable patch antenna achieved by the gravity-driven liquid metal was proposed, which obtained a working bandwidth of 3.69–4.95 GHz [21].

However, in some applications, a single-frequency-band antenna cannot meet the actual needs [22]. In the working procedures of these liquid-metal frequency-reconfigurable antennas introduced above, the antennas have only one resonant frequency when they are not deformed, so the antennas can only be used in a single-frequency band in a certain state. 

In this study, we report a dual-frequency-reconfigurable antenna, in which EGaIn was filled into the U-shaped glass pipe. The EGaIn was driven by a precise syringe pump to generate the height difference of the liquid metal in the two branches. After the structure of the U-shaped tube was selected, the EGaIn was injected to allow the initial heights of the EGaIn in the two branches were equal (10 mm). Then, the height of one of the branches was adjusted from 10 mm to 18 mm, meaning in that the two branches can obtain ultrawide working frequencies of 2.27–4.98 GHz (measured) and 2.71–8.58 GHz (measured), respectively. The antenna exhibited omnidirectional radiation characteristics with a maximum gain of 4.00 dBi (measured). The measurement results of S-parameters and gain patterns were in good agreement with the simulation results, which proved that the proposed antenna worked well. This research is a good attempt to design a multifrequency-reconfigurable antenna using liquid metal. This method of controlling the height change of multiple liquid-metal branches can also be used to further broaden the working bandwidth of the antenna.

## 2. Antenna Design

**Figure 1:**
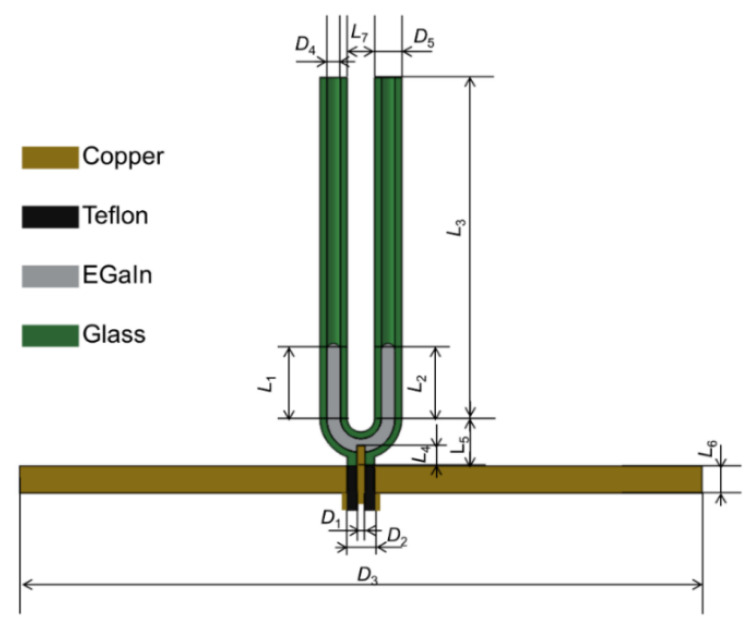
Front cross-sectional view of the antenna.

The structure of the antenna is presented in Figure 1. The antenna consisted of four parts: the subminiature version A (SMA) connector, a ground plane, a U-shaped glass tube, and a pressure supply device. The ground plane was made of copper, and the U-shaped glass tube was inserted into the middle hole of the ground plane. The inner needle of the SMA connector passed through the hole in the middle of the ground plane and was inserted into the hole at the bottom of the U-shaped glass tube as well as made contact with the liquid metal in the tube. The relative dielectric constant ε of glass is 3.7, and the dielectric loss tanδ is 0.0003 [23]. The liquid metal used in this work was a nontoxic gallium–indium alloy EGaIn (75.5% gallium, 24.5% indium) with a melting point of 15.5 °C and electrical conductivity of 3.46 × 10^6^ S/m [24]. To eliminate the oxide film of EGaIn, 0.5 mol/L NaOH solution was used to wet the U-shaped glass tube before injecting EGaIn into the tube [25].

**Figure 2:**
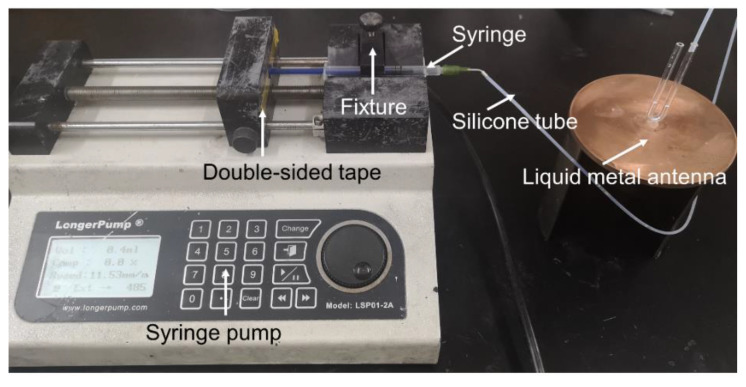
The antenna and the pressurizing device (a syringe pump).

The pressure can be supplied by, for example, a syringe, various mechanical pumps, an air pump, a syringe pump, and so on. As shown in Figure 2, a precise syringe pump (model: LSP01-2A, made by Longer Precision Pump Co., Ltd., Baoding, China) was adopted. The silicone tube was used to connect the syringe on the syringe pump to the right branch of the U-shaped tube. The scale with millimeter precision was engraved on the glass tube by a laser. The syringe pump controlled the air pressure in the tube, which could quickly and accurately control the height of the EGaIn.

**Figure 3:**
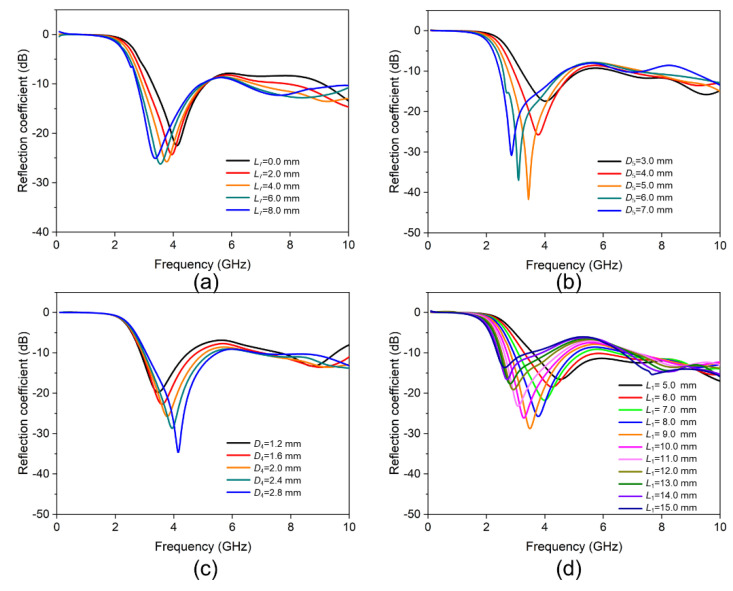
The impact of geometric parameters on the reflection coefficients of the antenna: (**a**) *L*_7_, (**b**)*D*_5_, (**c**) *D*_4_, and (**d**) *L*_1_.

For this antenna, the distance *L*_7_ between the two branches, the inner diameter *D*_4_, and the outer diameter *D*_5_ of the glass tube are all very sensitive dimensions. The initial geometric dimensions in Figure 1 were optimized by the high-frequency structure simulator (HFSS) software. Figure 3 shows the effect of changes in some geometric parameters on the reflection coefficient of the antenna. Figure 3a describes the change curve of the reflection coefficient of the antenna with frequency when the distance between the two branches *L*_7_ increased from 0 to 8 mm, and the outer diameter *D*_5_ = 4.0 mm, and the inner diameter *D*_4_ = 2.0 mm. During this process, the resonant frequency of the antenna dropped a little, and the minimum value of the reflection coefficient also did not change much. As shown in Figure 3b, when the distance between the two branches *L*_7_ = 4.0 mm and the inner diameter *D*_4_ = 2.0 mm, the process of increasing the outer diameter *D*_5_ from 3 mm to 7 mm would cause the reflection coefficient to first decrease and then increase. At the same time, the resonant frequency was also moving to the low frequency. Figure 3c shows the change curve of the reflection coefficient of the antenna when the distance between the two branches *L*_7_ = 4.0 mm and the outer diameter *D*_5_ = 4.0 mm, and the inner diameter *D*_4_ gradually increased from 1.2 mm to 2.8 mm. Obviously, the minimum value of the reflection coefficient increased, and the resonant frequency decreased. Figure 3d shows the change in reflection coefficient when the size of the U-shaped tube remained unchanged and the initial volume of the liquid metal increased (the initial height of *L*_1_ increased). When *L*_1_ increased, the reflection coefficient of the antenna first decreased and then increased, and the impedance bandwidth increased. When *L*_1_ was selected among the values of 8 mm, 9 mm, and 10 mm, the corresponding reflection coefficients of the antenna were the smallest ones. The optimized size adopted in this study was listed in Table 1 through numerical simulation.

## 3. Results and Discussion

The reflection coefficient of the antenna was measured by a vector network analyzer (VNA) E5063A (Keysight Technologies, Inc., Santa Rosa, DE, USA), and the gain pattern of the antenna was tested in the microwave anechoic chamber of the University of Electronic Science and Technology of China. Considering that the initial amount of liquid metal would affect the performance of the antenna, two cases were studied as an illustration, where the initial heights of *L*_1_ were 10 mm and 12 mm, respectively. A syringe was used to change the initial volume of the liquid metal.

The antenna reconstruction process was as follows: when slowly increased the pressure on the right branch, *L*_2_ slowly decreased while *L*_1_ slowly increased. The change process was continuous. Since the total amount of liquid metal was constant, the sum of *L*_1_ and *L*_2_ did not change. 

**Figure 4:**
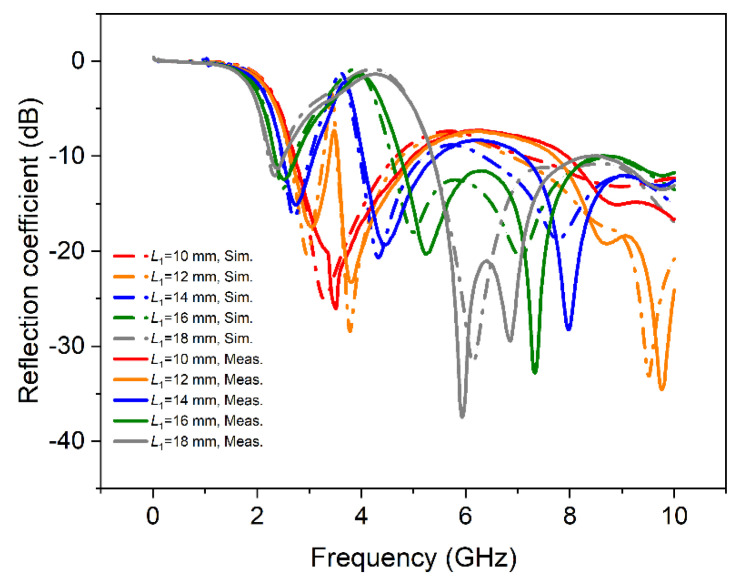
The reflection coefficient of the antenna (the initial condition: *L*_1_ = *L*_2_ = 10 mm).

As shown in Figure 4, when *L*_1_ = *L*_2_ = 10 mm, there was only one frequency band that satisfied the condition that S_11_ was less than −10 dB. The simulated and measured impedance bandwidths were 2.71–4.71 GHz (resonant at 3.28 GHz) and 2.71–4.98 GHz (resonant at 3.51 GHz), respectively. However, when *L*_1_ = 12 mm and *L*_2_ = 8 mm, the simulation results of the antenna had two frequency bands with center frequencies at 2.96 GHz and 3.78 GHz, and the corresponding measurements were 3.03 GHz and 3.79 GHz, respectively. The smaller of the two center frequencies was denoted as *f*_L_, and the larger one was denoted as *f*_H_. As the difference between *L*_1_ and *L*_2_ increased, and so did the difference between *f*_L_ and *f*_H_. Therefore, in Figure 4, as *L*_1_ increased, the two center frequencies of the antenna moved in two different directions away from the center frequency of the initial height of *L*_1_ (10 mm). When *L*_1_ = 18 mm and *L*_2_ = 2 mm, the reflection coefficient at *f*_L_ had increased to −11 dB. Thereafter, the continued increase in *L*_1_ was not conducive to the antenna, so *L*_1_ was no longer increased. Under the condition of *L*_1_ = 18 mm, the matching at *f*_H_ was also good, and even broadband characteristics could be maintained, as shown in Figure 4.

**Figure 5:**
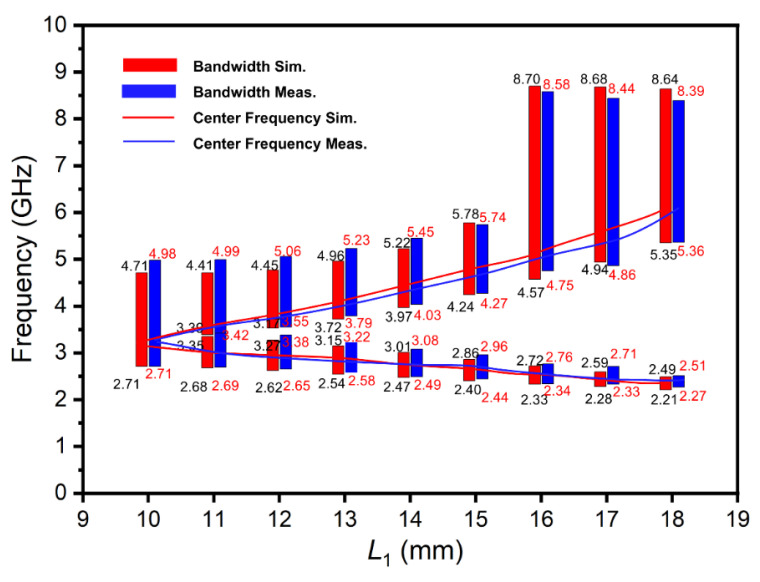
The bandwidth and center frequency of the antenna (the initial condition: *L*_1_ = *L*_2_ = 10 mm).

In Figure 5, the movement of the center frequency in both directions was obvious as *L*_1_ increased. In the process of increasing *L*_1_ from 10 mm to 18 mm, the antenna had two simulated tunable working frequency bands of 2.21–4.71 GHz and 2.71–8.70 GHz, respectively. The corresponding measurement data were 2.27–4.98 GHz and 2.71–8.58 GHz. It could be concluded from Figure 4 and Figure 5 that the designed U-shaped monopole antenna had dual-frequency characteristics when the heights of liquid metal in two branches were unequal. Additionally, the measuring and simulating results were consistent, which proved the validity of the antenna design.

**Figure 6:**
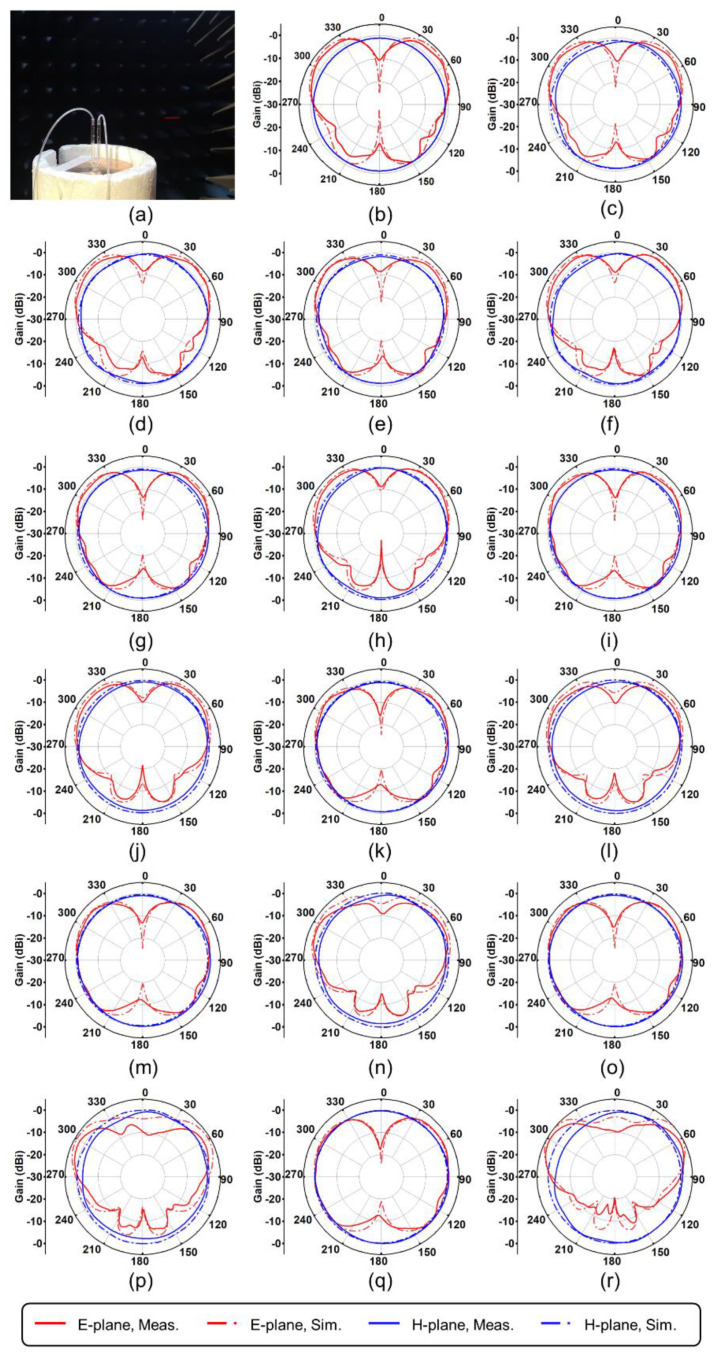
Radiation patterns of E-plane and H-plane (the initial condition: *L*_1_ = *L*_2_ = 10 mm). (**a**) The measurement environment; (**b**) *L*_1_ = *L*_2_ = 10 mm, *f*_L_ = *f*_H_ = 3.28 GHz; (**c**) *L*_1_ = 11 mm, *f*_L_ = 3.12 GHz; (**d**) *L*_1_ = 11 mm, *f*_H_ = 3.52 GHz; (**e**) *L*_1_ = 12 mm, *f*_L_ = 2.96 GHz; (**f**) *L*_1_ = 12 mm, *f*_H_ = 3.78 GHz; (**g**) *L*_1_ = 13 mm, *f*_L_ = 2.80 GHz; (**h**) *L*_1_ = 13 mm, *f*_H_ = 4.04 GHz; (**i**) *L*_1_ = 14 mm, *f*_L_ = 2.70 GHz; (**j**) *L*_1_ = 14 mm, *f*_H_ = 4.32 GHz; (**k**) *L*_1_ = 15 mm, *f*_L_ = 2.60 GHz; (**l**) *L*_1_ = 15 mm, *f*_H_ = 4.62 GHz; (**m**) *L*_1_ = 16 mm, *f*_L_ = 2.50 GHz; (**n**) *L*_1_ = 16 mm, *f*_H_ = 5.00 GHz; (**o**) *L*_1_ = 17 mm, *f*_L_ = 2.42 GHz; (**p**) *L*_1_ = 17 mm, *f*_H_ = 5.48 GHz; (**q**) *L*_1_ = 18 mm, *f*_L_ = 2.34 GHz; (**r**) *L*_1_ = 18 mm, *f*_H_ = 6.14 GHz.

Figure 6 describes the radiation patterns of the E-plane and H-plane on the *f*_H_ and *f*_L_ of the antenna under the initial conditions of *L*_1_ = *L*_2_ = 10 mm. The results indicated that the antenna exhibited very good omnidirectional radiation characteristics at *f*_L_. The antenna had a pair of small backlobes and a higher gain at *f*_H_.

The performance of the antenna (the initial condition: *L*_1_ = *L*_2_ = 10 mm) is listed in Table 2. When *L*_1_ = 18 mm and *f*_H_ = 6.14 GHz, the antenna had a maximum simulated gain of 5.44 dBi, and the corresponding measurement result was 4.00 dBi. The simulated maximum average efficiency (*E*_Ave., Simu. =_ 95%) was obtained when *L*_1_ = 16 mm and *f*_H_ = 5.00 GHz. When *L*_1_ = 18 mm, *f*_L =_ 2.34 GHz, the antenna obtained the maximum average efficiency (*E*_Ave., Meas._ = 86%) by measured. The measured efficiencies of the antenna were lower than the simulated ones due to manufacturing errors, measurement errors, and frequency offsets. 

**Figure 7:**
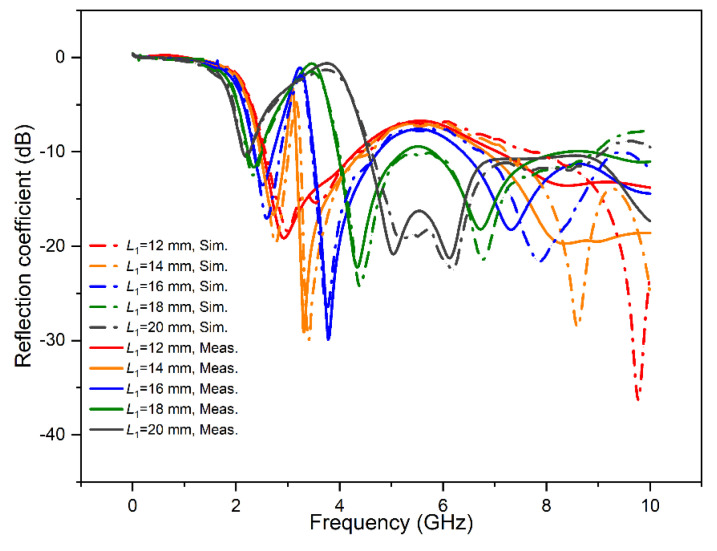
The reflection coefficient of the antenna (the initial condition: *L*_1_ = *L*_2_ = 12 mm).

**Figure 8:**
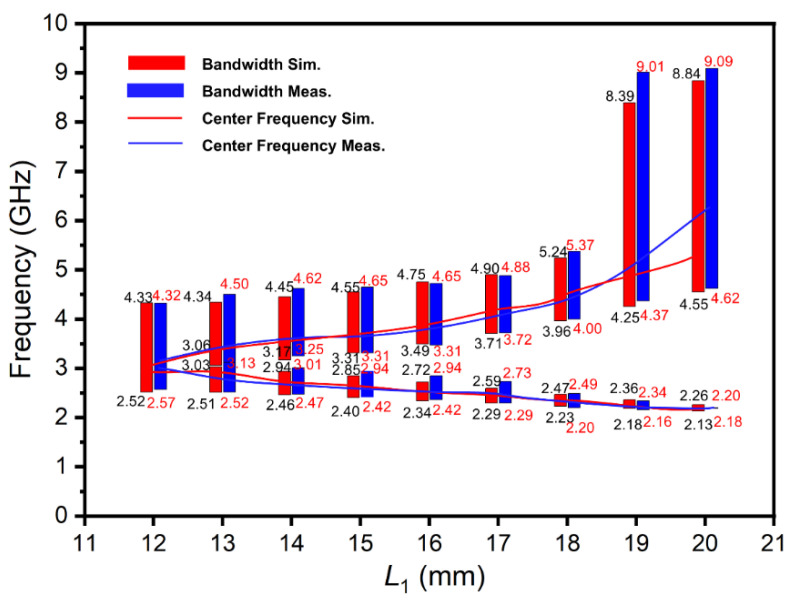
The bandwidth and center frequency of the antenna (the initial condition: *L*_1_ = *L*_2_ = 12 mm).

According to the theory of monopole antenna, the initial heights (*L*_1_ and *L*_2_) of the two elements of the antenna could affect the tuning range of the antenna. The larger the initial height of *L*_1_, the lower the *f*_L_ that could be obtained, and the smaller the initial height of *L*_2_, the larger the *f*_H_ that could be obtained. For this reason, the case of the initial heights *L*_1_ = *L*_2_ = 12 mm was also studied, which was expected to obtain a lower *f*_L_. Similar as in the above case, Figure 7 and Figure 8 show that the reflection coefficient and impedance bandwidth, respectively, change with *L*_1_. It can be seen from Figure 7 that increasing *L*_1_ from 12 mm to 20 mm (which could be regarded as the process of antenna reconstruction), the S_11_ of the antenna at *f*_L_ increased significantly. It is also indicated in Figure 8 that during the antenna reconstruction process, the two branches of the antenna corresponded to two operating frequency bands. The simulation data were 2.13–4.33 GHz and 2.52–8.84 GHz meanwhile the experimental results were 2.18–4.32 GHz and 2.57–9.09 GHz, respectively. The downward trend of *f*_L_ was in line with expectations, and the simulation and measuring results were in good agreement.

**Figure 9:**
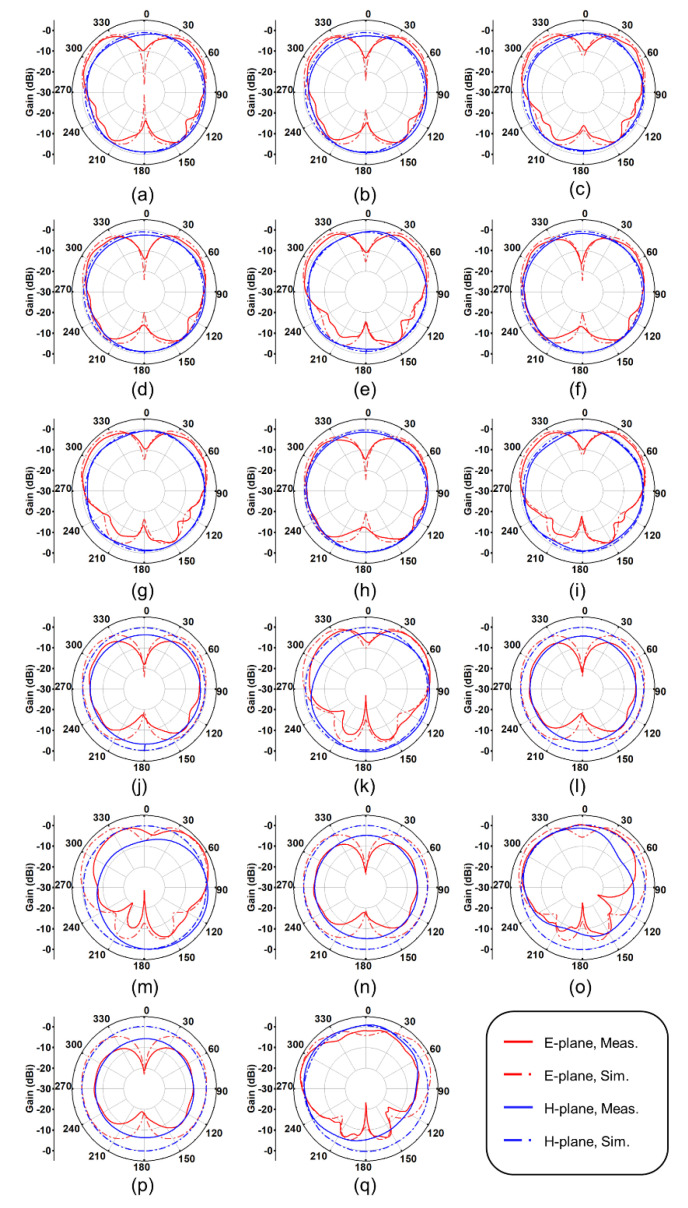
Radiation patterns of E-plane and H-plane (the initial condition: *L*_1_ = *L*_2_ = 12 mm). (**a**) *L*_1_ = *L*_2_ = 12 mm, *f*_L_ = *f*_H_ = 2.92 GHz; (**b**) *L*_1_ = 13 mm, *f*_L_ = 2.84 GHz; (**c**) *L*_1_ = 13 mm, *f*_H_ = 3.12 GHz; (**d**) *L*_1_ = 14 mm, *f*_L_ = 2.72 GHz; (**e**) *L*_1_ = 14 mm, *f*_H_ = 3.32 GHz; (**f**) *L*_1_ = 15 mm, *f*_L_ = 2.62 GHz; (**g**) *L*_1_ = 15 mm, *f*_H_ = 3.54 GHz; (**h**) *L*_1_ = 16 mm, *f*_L_ = 2.52 GHz; (**i**) *L*_1_ = 16 mm, *f*_H_ = 3.78 GHz; (**j**) *L*_1_ = 17 mm, *f*_L_ = 2.42 GHz; (**k**) *L*_1_ = 17 mm, *f*_H_ = 4.06 GHz; (**l**) *L*_1_ = 18 mm, *f*_L_ = 2.34 GHz; (**m**) *L*_1_ = 18 mm, *f*_H_ = 4.34 GHz; (**n**) *L*_1_ = 19 mm, *f*_L_ = 2.26 GHz; (**o**) *L*_1_ = 19 mm, *f*_H_ = 4.66 GHz; (**p**) *L*_1_ = 20 mm, *f*_L_ = 2.20 GHz; (**q**) *L*_1_ = 20 mm, *f*_H_ = 5.04 GHz.

The radiation patterns of E-plane and H-plane obtained by simulation and measurement under the initial condition of *L*_1_ = *L*_2_ = 12 mm are showcased in Figure 9. Similar to the results of the above group in Figure 6, the antenna had a larger peak gain at *f*_H_, and a smaller peak gain at *f*_L_. The maximum gain obtained by the simulation was 3.67 dBi (*L*_1_ = 14 mm, at 3.32 GHz), and the maximum gain obtained by the test was 3.72 dBi (*L*_1_ = 18 mm, at 4.39 GHz). Overall, the measurement and simulation results had a consistent trend. The measured gains of the last four cases in Figure 9 were smaller than the simulated gains. This was because under the condition of the large frequency offset, the measured gains might not correspond to the center frequency of the antenna. 

Similarly, the performances of the antenna after the initial height of *L*_1_ was adjusted to 12 mm are listed in Table 3. When *L*_1_ = 20 mm, *f*_H_ = 5.04 GHz, the antenna had the maximum average efficiency (*E*_Ave., Simu._ = 95%). The maximum average efficiency (*E*_Ave., Meas._ = 75%) was obtained by measuring the antenna in multiple states.

Table 4 lists the performance comparison of some solid antennas and liquid-metal frequency-reconfigurable antennas previously reported with this work. Different from the common solid-state antennas [3,26,27], no semiconductor devices were used in the liquid-metal frequency-reconfigurable antennas, which could avoid nonlinear problems. Compared with the previous reconfigurable liquid-metal antennas [14,16,17,19,20,21], only this work had dual-frequency-reconfigurable characteristics. The two branches had 2.27–4.98 GHz and 2.71–8.58 GHz ultrawide working frequency bands (the initial condition: *L*_1_ = *L*_2_ = 10 mm), respectively. At the same time, the antenna peak gain reached 4.00 dBi (measured). In another geometric height (the initial condition: *L*_1_ = *L*_1_ = 12 mm), the two branches had two ultrawide tunable working frequency bands of 2.18–4.32 GHz and 2.57–9.09 GHz, respectively. Based on the design of this study, a series of reconfigurable antennas with different working frequency bands could be obtained just by adjusting the initial amount of liquid metal.

**Figure 10:**
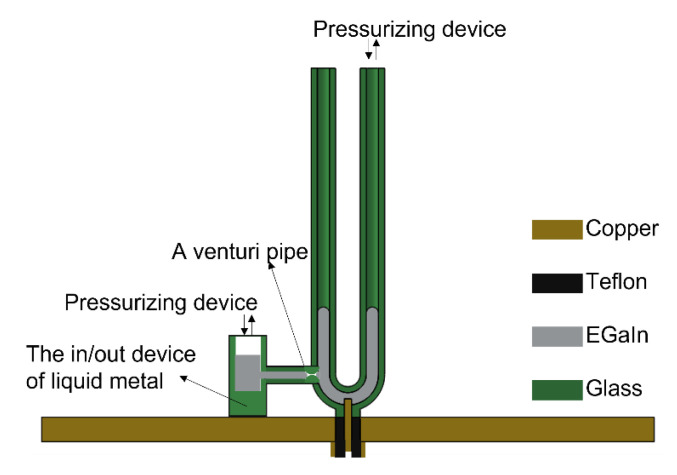
A more optimized structure of this antenna.

However, based on the current design, it might be inconvenient to change the initial amount of liquid metal during the reconfiguration process of the antenna, since the sum of the heights of the liquid metal in the two branches is a constant. This would inevitably lead to that the two resonant frequencies of the antenna being interrelated and restricting each other. To realize the independent reconstruction of dual frequency, an improved antenna structure was proposed, as shown in Figure 10. Based on the original structure, an in/out device of liquid metal was added. Because liquid metal has a high surface tension, a venturi pipe between the U-shaped tube and the in/out device of liquid metal would isolate the liquid metal in both sides. According to the sum of the heights of the liquid metal in the two branches required for reconstruction, this in/out device of liquid metal was applied to adjust the volume of the liquid metal in the U-shaped tube. In this way, the sum of the heights of the liquid metal in the two branches would be no longer a constant, then the two resonant frequencies could be adjusted independently, and the reconfigurable bandwidth of the antenna would also be greatly expanded. In fact, the antennas in this study can also be designed as planar structures in the future by using a U-shaped slot filled with liquid metal. The improved antenna can be fed by a coplanar waveguide, which will facilitate antenna and circuit integration.

## 4. Conclusions

In this study, a U-shaped dual-frequency-reconfigurable liquid-metal monopole antenna was designed and analyzed. When the initial height of *L*_1_ was 10 mm, the two branches could, respectively, obtain two working frequency bands of 2.27–4.98 GHz and 2.71–8.58 GHz (measured), and the measured maximum gain was 4.00 dBi via adjusting *L*_1_ in the range of 10–18 mm. Considering the effects of initial heights of *L*_1_ and *L*_2_ on the working bandwidth, the case in which the initial heights of *L*_1_ and *L*_2_ were both set as 12 mm was also investigated. Two ultrawide tunable working frequency bands of 2.18–4.32 GHz and 2.57–9.09 GHz (measured) could be obtained by adjusting *L*_1_ in the range of 12–20 mm. For this case, the measured maximum gain was 3.72 dBi. The simulated and measured maximum average efficiencies were 95% and 86%, respectively. This bandwidth includes multiple frequency bands such as wireless local area networks (2.4 GHz and 5 GHz) and 5G communications (n7, n38, n41, n77, n78 and n79). It was indicated that, based on the design presented in this study, a series of multifrequency-reconfigurable antennas with different working frequency bands could be obtained by adjusting the height of the liquid metal in the two branches, which might have broad application prospects in fields such as navigation and base stations.

## Figures and Tables

**Table 1:** Dimensions of the Optimized Antenna (Unit: mm).

*D*_1_	*D*_2_	*D*_3_	*D*_4_
1.2	4.0	100	2.0
*D*_5_	*L*_1_	*L*_2_	*L*_3_
4.0	10.0	10.0	50
*L*_4_	*L*_5_	*L*_6_	*L*_7_
4.0	8.0	4.0	4.0

**Table 2:** The performances of the antenna (the initial condition: *L*_1_ = *L*_2_ = 10 mm).

The Length of *L*_1_ (mm)		Center Frequency (Sim., GHz)	Center Frequency (Meas., GHz)	Impedance Bandwidth (Sim.,GHz)	Impedance Bandwidth (Meas., GHz)	Peak Gain (Sim., dBi)	Peak Gain (Meas., dBi)	Average Effiency (Sim., %)	Average Effiency (Meas., %)
10	*f*	3.28	3.51	2.71–4.71	2.71–4.98	3.72	3.20	84	76
12	*f*_L_	2.96	3.03	2.62−3.27	2.65–3.38	3.20	3.60	88	79
	*f*_H_	3.78	3.79	3.53–4.77	3.55–5.06	4.04	3.45	83	73
14	*f*_L_	2.70	2.74	2.47–3.01	2.49–3.08	2.49	2.55	82	81
	*f*_H_	4.32	4.42	3.97–5.22	4.03–5.45	3.79	2.33	93	73
16	*f*_L_	2.50	2.51	2.33–2.72	2.34–2.76	2.03	2.06	85	83
	*f*_H_	5.00	5.24	4.57–8.70	4.75–8.58	3.52	2.17	95	68
18	*f*_L_	2.34	2.39	2.21–2.49	2.27–2.51	1.67	1.78	88	86
	*f*_H_	6.14	5.93	5.35–8.64	5.36–8.39	5.44	4.00	93	74

**Table 3:** The performances of the antenna (the initial condition: *L*_1_ = *L*_2_ = 12 mm).

The length of *L*_1_ (mm)		Center Frequency (Sim., GHz)	Center Frequency (Meas., GHz)	Impedance Bandwidth (Sim.,GHz)	Impedance Bandwidth (Meas., GHz)	Peak Gain (Sim., dBi)	Peak Gain (Meas., dBi)	Average Effiency (Sim., %)	Average Effiency (Meas., %)
12	*f*	2.92	2.98	2.52–4.33	2.57–4.32	3.11	2.60	83	75
14	*f*_L_	2.72	2.76	2.46–2.94	2.47–3.01	2.51	1.53	82	70
	*f*_H_	3.32	3.41	3.17–4.45	3.25–4.62	3.67	2.38	84	73
16	*f*_L_	2.52	2.59	2.34–2.72	2.36–2.85	1.96	1.40	85	75
	*f*_H_	3.78	3.75	3.49–4.75	3.47–4.72	3.57	2.58	88	75
18	*f*_L_	2.34	2.33	2.23–2.47	2.20–2.49	1.52	−2.73	88	33
	*f*_H_	4.34	4.39	3.96–5.24	4.00–5.37	3.32	3.72	94	63
20	*f*_L_	2.20	2.19	2.13–2.26	2.18–2.20	1.26	−4.36	91	22
	*f*_H_	5.04	6.19	4.55–8.84	4.62–9.09	3.40	2.71	95	63

**Table 4:** Performance comparison for different antenna types.

Ref.	Radiator	Methods of Reconstruction	Reconfigurable Types	Max Size (mm)	Working Bandwidth (Meas.)	Peak Gain (Meas.)
[1]	Planar inverted-F	RF-MEMS	Frequency	104	0.718 (2.6%) and 4.96 (7.6%)	3.3 dBi
[2]	Slot	PIN diodes	Frequency	43	6.0–10.6 GHz	3.2 dBi
[3]	Patch	Varactor diodes	Frequency	100	1.92–2.51 GHz	5.91 dBi
[16]	Monopole	Liquid metal	Frequency	>75	0.66–3.4 GHz	3.40 dBi
[17]	Monopole	Liquid metal	Frequency	152.4	1.29–5.17 GHz	>1.3 dBi
[19]	Quasi-Yagi	Liquid metal	Frequency	88	1.8–2.4 GHz	8.5 dBi
[20]	Monopole	Liquid metal	Frequency	100	1.25–2.00 GHz	2.90 dBi
[21]	Patch	Liquid metal	Frequency	40	3.69–4.95 GHz	1.43 dBi
[28]	Slot	Liquid metal	Frequency	>60	1.41–1.84 GHz	4.8 dBi
[29]	Magnetoelectric Dipole	Liquid metal	Frequency	100	1.79–3.85 GHz	9.4 dBi
This work	U-shape Monopole	Liquid metal	Dual frequency	100	2.27–4.98 GHz and 2.71–8.58 GHz; 2.18–4.32 GHz and 2.57–9.09 GHz	4.00 dBi; 3.72 dBi

## Data Availability

Data are contained within the article.

## References

[1] Zohur A., Mopidevi H., Rodrigo D., Unlu M., Jofre L., Cetiner B.A. (2013). RF MEMS Reconfigurable Two-Band Antenna. IEEE Antennas Wirel. Propag. Lett..

[2] Pazin L., Leviatan Y. (2013). Reconfigurable Slot Antenna for Switchable Multiband Operation in a Wide Frequency Range. IEEE Antennas Wirel. Propag. Lett..

[3] Gu H., Wang J., Ge L. (2015). Circularly Polarized Patch Antenna with Frequency Reconfiguration. IEEE Antennas Wirel. Propag. Lett..

[4] Zou H., Wang W., Zhang G., Qin F., Tian C., Yan Y. (2016). Experimental investigation on an integrated thermal management system with heat pipe heat exchanger for electric vehicle. Energy Convers. Manag..

[5] Yan J., Lu Y., Chen G., Yang M., Gu Z. (2018). Advances in liquid metals for biomedical applications. Chem. Soc. Rev..

[6] Daalkhaijav U., Yirmibesoglu O.D., Walker S., Mengüç Y. (2018). Rheological Modification of Liquid Metal for Additive Manufacturing of Stretchable Electronics. Adv. Mater. Technol..

[7] Wang X., Liu J. (2016). Recent Advancements in Liquid Metal Flexible Printed Electronics: Properties, Technologies, and Applications. Micromachines.

[8] Varga M., Ladd C., Ma S., Holbery J., Tröster G. (2017). On-skin liquid metal inertial sensor. Lab Chip.

[9] Sheng L., Zhang J., Liu J. (2014). Diverse Transformations of Liquid Metals Between Different Morphologies. Adv. Mater..

[10] Liu Y., Wang Q., Jia Y., Zhu P. (2020). A Frequency- and Polarization-Reconfigurable Slot Antenna Using Liquid Metal. IEEE Trans. Antennas Propag..

[11] Zhang T., Chen Y., Yang S. A Frequency and Polarization Reconfigurable Spiral Antenna based on Liquid Metal. Proceedings of the 2021 International Applied Computational Electromagnetics Society (ACES-China) Symposium.

[12] Baig M.U., Elassy K.S., Høst-Madsen A., Ohta A.T., Shiroma W.A., Nosratinia A. (2021). Leveraging discrete modulation and liquid metal antennas for interference reduction. EURASIP J. Wirel. Commun. Netw..

[13] Zhou Y., Fang S., Liu H., Wang Z., Shao T. (2020). A Function Reconfigurable Antenna Based on Liquid Metal. Electronics.

[14] Kubo M., Li X., Kim C., Hashimoto M., Wiley B.J., Ham D., Whitesides G.M. (2010). Stretchable Microfluidic Radiofrequency Antennas. Adv. Mater..

[15] Gough R.C., Morishita A.M., Dang J.H., Hu W., Shiroma W.A., Ohta A.T. (2014). Continuous Electrowetting of Non-toxic Liquid Metal for RF Applications. IEEE Access.

[16] Wang M., Trlica C., Khan M.R., Dickey M.D., Adams J.J. (2015). A reconfigurable liquid metal antenna driven by electrochemically controlled capillarity. J. Appl. Phys..

[17] Dey A., Guldiken R., Mumcu G. (2016). Microfluidically Reconfigured Wideband Frequency-Tunable Liquid-Metal Monopole Antenna. IEEE Trans. Antennas Propag..

[18] Huff G.H., Pan H., Hartl D.J., Frank G.J., Bradford R.L., Baur J.W. (2017). A Physically Reconfigurable Structurally Embedded Vascular Antenna. IEEE Trans. Antennas Propag..

[19] Shah S.I.H., Lim S. (2018). Microfluidically Frequency-Reconfigurable Quasi-Yagi Dipole Antenna. Sensors.

[20] Qin P., Wang L., Liu T.-Y., Wang Q.-Y., Fu J.-H., Huang G.-L., Gui L., Liu J., Deng Z.-S. (2021). The Design and Manufacturing Process of an Electrolyte-Free Liquid Metal Frequency-Reconfigurable Antenna. Sensors.

[21] Qin P., Huang G.-L., Liang J.-J., Wang Q.-Y., Fu J.-H., Zhu X.-Y., Liu T.-Y., Gui L., Liu J., Deng Z.-S. (2021). A Gravity-Triggered Liquid Metal Patch Antenna with Reconfigurable Frequency. Micromachines.

[22] Jenath Sathikbasha M., Nagarajan V. (2020). Design of Multiband Frequency Reconfigurable Antenna with Defected Ground Structure for Wireless Applications. Wirel. Pers. Commun..

[23] Sergolle M., Castel X., Himdi M., Besnier P., Parneix P. (2020). Structural composite laminate materials with low dielectric loss: Theoretical model towards dielectric characterization. Compos. Part C Open Access.

[24] Liang Q., Yang Z., Guo J., Li Z., Chen T., Li D. (2020). A high-efficient tunable liquid metal-based electromagnetic absorbing metamaterial. J. Mater. Sci. Mater. Electron..

[25] Liu T., Sen P., Kim C. Characterization of liquid-metal Galinstan^®^ for droplet applications. Proceedings of the 2010 IEEE 23rd International Conference on Micro Electro Mechanical Systems (MEMS).

[26] Mansoul A., Ghanem F., Hamid M.R., Trabelsi M. (2014). A Selective Frequency-Reconfigurable Antenna for Cognitive Radio Applications. IEEE Antennas Wirel. Propag. Lett..

[27] Erdil E., Topalli K., Unlu M., Civi O.A., Akin T. (2007). Frequency Tunable Microstrip Patch Antenna Using RF MEMS Technology. IEEE Trans. Antennas Propag..

[28] Dang J.H., Gough R.C., Morishita A.M., Ohta A.T., Shiroma W.A. (2015). Liquid-metal frequency-reconfigurable slot antenna using air-bubble actuation. Electron. Lett..

[29] Su M., Geng X., Zhang Y., Wang A. (2021). Frequency-Reconfigurable Liquid Metal Magnetoelectric Dipole Antenna. IEEE Antennas Wirel. Propag. Lett..

